# Case-series of patients treated with anti-NMDAR encephalitis at Semmelweis University

**DOI:** 10.1192/j.eurpsy.2024.1427

**Published:** 2024-08-27

**Authors:** L. Herman, J. M. Réthelyi, I. Sipos, R. I. Zsigmond

**Affiliations:** ^1^Department of Psychiatry and Psychotherapy; ^2^Department of Neurology, Semmelweis University, Budapest, Hungary

## Abstract

**Introduction:**

Anti-NMDAR encephalitis is an autoimmune disorder, characterized by neuropsychiatric symptoms, such as mood instability, psychosis, catatonia, dyskinesia, seizures and vegetative lability. Psychiatric symptoms usually occur in the initial phase, therefore almost half of the patients are first observed at a psychiatric unit, however in later phases the patients’ condition often show progression with the characteristic neurological symptoms, such as perioral dyskinesia and seizures. Although, early recognition and treatment is essential to reach good outcomes, delay in the diagnostic process often happens due to the unspecific early symptoms and the lack of knowledge of this disorder amongst psychiatrists.

Moreover, there are cases, where neurological symptoms do not appear, which can lead to diagnostic failure and mismanagement of these patients. Since anti-NMDAR encephalitis is a rare condition, it is important to treat such cases in specific centres, where sufficient knowledge and multidisciplinary approaches are available.

**Objectives:**

Our aim was to gather all patients’ data treated with anti-NMDAR encephalitis at two departments (Neurology and Psychiatry) of Semmelweis University. We wanted to analyse psychiatric manifestations of the disorder in details and follow these symptoms long term, with special interest on the cognitive symptoms.

One of our aims was to follow-up these patients and measure antibody titres in their serum, to be able to asses, whether there was any association between prolonged serum positivity and cognitive impairment.

**Methods:**

We have retrospectively analysed data of previous cases and prospectively followed up recently hospitalised patients.

Neurocognitive assessment had been conducted by the same psychologist, all the patients were followed up by the same interdisciplinary team, including a neurologist and two psychiatrists. Laboratory tests (autoimmune antibody essays) were conducted by the Immunological Laboratory at Semmelweis University.

**Results:**

Altogether, 13 female patients were treated with anti-NMDAR encephalitis in the past ten years at Semmelweis University. All of them received plasma exchange, iv. steroids and azathioprine. 8 out of the 13 needed ventilation and intensive care treatment. 2 of these patients have mild psychiatric symptoms as residual symptoms, and 1 of them is still in the recovery stage, currently experiencing mild cognitive symptoms.

Only two patient had ovarian teratomas out of the 13, which is a lower number than expected from previous studies.

4 out of 12 had positive antibody titre at follow up, one patient is still at recovery stage, however her antibody titres are still very high.

**Conclusions:**

Semmelweis University is one of the largest centre treating patients with anti-NMDAR encephalitis in Hungary. We had altogether 13 patients in the last ten years, with very good outcome, since all of them recovered, although 2 have residual symptoms.

**Disclosure of Interest:**

None Declared

